# Exploring the Unique and Interactive Effects Between Callous-Unemotional and Autistic Traits with Parental Practices, Care, and Distress in a Community Sample

**DOI:** 10.1007/s10802-024-01222-9

**Published:** 2024-06-15

**Authors:** Giorgos Georgiou, Chara A. Demetriou, Kostas A. Fanti

**Affiliations:** Department of Social and Behavioral Sciences, European University Cyprus, P.O. Box 22006, 1516 Nicosia, CY Cyprus

**Keywords:** Autistic traits, Callous unemotional traits, Parental distress, Parental care, Parental practices

## Abstract

Parental practices and stress are associated with both CU and autistic traits, with parents of children with these traits facing challenges that other parents do not encounter. However, the majority of available studies focused mainly on the unique effects of CU and autistic traits with parental stress and practices without exploring their interaction. The current study examines the distinct associations and interactions between CU and autistic traits with parental practices (parental involvement, poor monitoring, inconsistent discipline, and corporal punishment), care, and distress after considering the effect of conduct problems (CPs), age and sex in a Greek-Cypriot sample (*N* = 146, *M*age = 7.30, *SD* = 1.43). Hierarchical multiple regression analysis revealed that children with CU traits were more likely to experience negative parenting, while parents showed heightened levels of distress. Notably, the study found no association between CU traits and positive parental practices. Further analysis indicated no significant relation between autistic traits and interactions with the target variables, signifying that these traits are not associated with difficulties in parenting and distress. No sex differences were found in all analyses. Age was negatively significant only in relation to parental distress These findings provide valuable insights into the impact of CU traits and underscore the need for additional studies investigating the impact of autistic traits, possibly within clinical samples.

## Introduction

Callous-unemotional (CU) and autistic traits are both related to emotional processing deficits (e.g., empathy components; Georgiou et al., 2019; Jones et al., 2010) and difficulties in social interaction (Sipes et al., 2011; Waller et al., 2013). CU traits refer to a group of characteristics related to callous use of others, lack of remorse or empathic concern, shallow or deficient emotions, and lack of concern about performance (Frick & White, 2008). Autistic traits on the other hand refer to a continuum of traits below the threshold of the diagnosis of Autism Spectrum Disorder, with impairments in the ability to understand others’ mental states and perspectives being among the core features of these traits (Baron-Cohen, 2000; Constantino & Todd, 2003; Hill & Frith, 2003).

Theoretical and empirical work link both traits with environmental (Crowell et al., 2019; Waller et al., 2013) and biological/ genetic (Bons et al., 2013; Moore et al., 2019; Persico & Napolioni, 2013) risk factors. Research suggests that parental practices, while not the root cause of these difficulties, can still have a detrimental effect. Negative parental practices, distress, and negative parent–child interactions as a response to children’s behavioral problems (e.g., Caspi & Moffitt, 1995; Huh et al., 2006; Kerr & Stattin, 2003; Muñoz et al., 2011) may influence the development of both CU and autistic traits (Berg-Nielsen et al., 2002; Crowell et al., 2019).

Despite evidence of distinct emotional and physiological profiles, empirical support of the co-occurrence between CU and autistic traits in children and adolescents is also evident (Leno et al., 2015, 2021; Pasalich et al., 2014). However, most prior work did not consider the interaction between CU and autistic traits in predicting parents’ reactions. Thus, aiming to inform the literature on the unique but also interactive effects of these traits, the current study was designed to examine the relation of CU, autistic traits, and their interactions with parental practices, care, and distress after accounting for levels of conduct problems (CPs), age and sex.

### Parental Practices in Relation to CU and Autistic Traits

Although all parents must adapt their parenting techniques and strategies according to the needs of their children, parents of children with CU and autistic traits face unique challenges. Challenges associated with these traits make the process of parenting even more demanding and complicated. Parental practices are behaviors toward offspring, including attitudes and reactions (Berg-Nielsen et al., 2002). Prior research examining the relation of psychopathic traits with parental practices highlighted the negative impact of CU traits on parent–child interactions. This line of work supported that children exhibiting high levels of CU traits tend to be insensitive to typical parental socialization practices (i.e., effective discipline strategies) (Waller et al., 2013) and lack concern over punishment (Hawes & Dadds, 2005; Pardini & Byrd, 2012). These characteristics may explain why parents of those children adopt ineffective practices such as inconsistent discipline, poor monitoring, and corporal punishment (Barker et al., 2011; Fanti et al., 2023; Georgiou et al., 2023). A prominent line in CU traits research has supported that children and adolescents characterized by CU traits are at high risk for severe and stable behavioral problems such as CPs (Fanti, 2018; Frick & White, 2008). Having a child who lacks empathy concern, and also exhibits CPs can be very distressing to parents, leading to dysfunctional parenting performance and even dissatisfaction with their role as parents (Fanti & Centifanti, 2014; Fite et al., 2008). However, Hawes et al. (2011) revealed that the relation between parental practices and CU traits was independent of associations with CPs and other environmental factors, proposing that CU traits uniquely accounted for the change in parental practices over time (i.e., increased level of inconsistent discipline and corporal punishment among older children only, and reduced levels of parental involvement).

Difficulties in social interaction and communication among children with autistic traits constitute a challenge for forming relationships with others, including parent–child relationships (Seltzer et al., 2000; Teague et al., 2018). Prior research revealed that less than 50% of children high on autistic traits can form close and secure relationships with their caregivers(for a review, see Teague et al., 2017). Several studies also investigated the relationship between autistic traits and parental practices, pointing to inconsistent findings. On the one hand, findings suggest that parental sensitivity, warmth behavior, responsiveness, and mutually sustained play did not significantly differ between parents of children with ASD and those without or with other difficulties (Baker et al., 2010; Kasari et al., 1988; Ku et al., 2019). On the contrary, Blacher and colleagues (2013) revealed that mothers of children with ASD engaged in more negative parental behaviors compared to mothers of typically developing children. Similarly, additional work suggests that mothers of children with ASD are less likely to stimulate development through positive parenting or disciplining their child compared to mothers of children without ASD (Lambrechts et al., 2015). Moreover, the level of severity of autistic traits can explain individual differences in parental reactions. Specifically, increased severity adversely affected communication, emotional expression, responsivity, and mood (Beurkens et al., 2013). Importantly, a recent meta-analysis proposed that what seems to differ between parents of children with ASD compared to parents of children without ASD is the level of parental control and negativity dimensions (i.e., aversion, hostility, and overt communication of negative feelings) (Ku et al., 2019). Despite the inconsistency between results, one factor was stable and affected in the same way parents of children with ASD and autistic traits –parental distress.

### Parental Distress in Relation to CU and Autistic Traits

Parental distress is a complex process involving emotional, behavioral, and cognitive components that are directly related and affect parents’ appraisal of their role, their psychological well-being, and the qualities of the child-parent relationship (Deater-Deckard, 1998; Whiteside-Mansell et al., 2007). The stable and long-lasting difficulties of children with CU and autistic traits involve many demands and challenges beyond those of parenting typically developing children (for autistic traits and autism, see: Enea & Rusu, 2020; for CU traits, see: Frick & White, 2008). Regarding CU traits, findings support that parents feel more dissatisfied with their role and their parenting performance if their child experiences behavioral problems and a callous and emotionless profile (Fanti et al., 2017; Fanti & Centifanti, 2014; Fite et al., 2008). Characteristics such as fearlessness, low sensitivity to punishment, and an unemotional profile may be perceived by parents as particularly distressing (Fanti & Centifanti, 2014) and may also lead to harsh parenting and conflict between parents and children (Fanti et al., 2023). Thus, it is possible that not the behavioral problems per se but their co-occurrence with a more callous profile can lead to parental distress. In line with this explanation, Buodo and colleagues (2013) revealed that parents of children engaging in antisocial behaviors experience a higher level of parental distress in cases where their child experiences a physiological under-reactivity profile – a profile linked to CU traits—in contrast to children with high levels of physiological reactivity. Moving to therapy, Somech and Elizur (2012), while evaluating a co-parent training program, found that reductions in CU traits were associated with improvements in parental distress, highlighting the strong relation between these two factors.

Similarly, parents of children with autism are more likely to experience higher levels of stress not only in comparison to parents of children without developmental or emotional difficulties but also compared to children with difficulties other than autism (Rao & Beidel, 2009; Schieve et al., 2007). There are several reasons for this relation, such as social difficulties, communication deficits, and problem behaviors, as well as additional family factors like the frequent use of childcare services, difficulties in employment, limited time for family activities, etc. (Montes & Halterman, 2008; Rao & Beidel, 2009). Also, the severity of difficulties, and especially the co-occurrence with aggression and antisocial behavior, is associated with stable high levels of parental distress in the long term, in contrast to families with the absence of such maladaptive behaviors (Gray, 2002). In a recent review, Enea and Rusu (2020) highlighted that children's emotional and behavioral problems are the best predictors of parenting stress in parents of children with ASD or autistic traits, a profile that may match the concept of interaction between autistic and CU traits – something that the current study aimed to address.

### Current Study

Although several studies pointed to the importance of accounting for the interaction between CU and autistic traits for understanding emotion recognition and empathy deficits (e.g., Georgiou et al., 2019; Leno et al., 2021; Pasalich et al., 2014; Rogers et al., 2006), proposing a so-called “double-hit,” only limited work investigated the interactive effects between autistic and CU traits in relation to parental outcomes. If the interaction of these two traits can lead to a different and, in some cases, more severe profile, then it is important to investigate the effects on their environment – namely parents – and especially parental practices and distress. Thus, the current study has two primary aims: First, to investigate the unique effects of the two traits with parental practices and distress after controlling for the impact of CPs, sex and age. Secondly, to investigate if the interaction of these two traits can lead to different difficulties in parental practices and higher levels of parenting distress.

Based on previous findings, we expected that CU traits would be associated with increased negative parental practices and decreased positive parental practices, such as parental involvement and care (Barker et al., 2011; Fanti et al., 2023; Georgiou et al., 2023). Regarding autistic traits, it is proposed that due to impairments in social communication and interaction, a link with more negative parental practices will be revealed (Blacher et al., 2013; Lambrechts et al., 2015). By testing for the interaction between autistic and CU traits, we might be able to explain the inconsistency of previous findings. In general, we expected our findings to provide new evidence of how each trait would be uniquely related to positive and negative parental practices. At the same time, controlling for CP will highlight the effects of these traits on parental behavior over and above their behavioral problems. Concerning parental distress, both traits were expected to be related to increased parental distress, which might be explained by their emotional and interpersonal difficulties (Fanti & Centifanti, 2014; Rao & Beidel, 2009; Schieve et al., 2007). The combination of high CU and autistic traits was anticipated to impose more severe difficulties in parents’ attempts for consistent parenting, engagement with more negative parental practices, and increased levels of parental distress.

In addition, we also investigated and controlled for age and sex differences in the relation of CU, autistic traits, and their interaction. As far as we know, few empirical studies on CU and autistic traits in girls are available (Colins et al., 2014a, b; 2012; Goldenfeld et al., 2005). Concerning parenting, only one study proposed that sex moderates the role of parenting on CU traits (Hawes et al., 2011). Specifically, it was revealed that positive parenting changed CU traits levels, mainly in girls, and parental involvement, mainly in boys. Regarding autistic traits, no studies investigated sex differences in parental practices. Focusing on parental distress, few studies have examined gender differences in autistic traits, with the results being inconsistent – some proposing no differences in parental distress (Postorino et al., 2015) and some suggesting higher parenting distress for parents of girls (Zamora et al., 2014). For CU traits, as far as we know, there are no studies investigating sex in relation to parental distress.

## Methods

### Participants and Procedure

The current study was based on parent reports of their children’s characteristics and their perception regarding parental care, distress, and practices. The sample consisted of 146 kindergarten and primary school children living in the Republic of Cyprus, roughly equally divided between boys and girls (56.2% *boys*; *n* = 82). Children ranged in age from 4–10 years (*M*age = 7.30, *SD* = 1.43). No participants were reported having a mental health diagnosis or diagnosed with specific conditions. Following approval of the study by the Centre of Educational Research and Assessment (CERE) of Cyprus, the Pedagogical Institute, the Ministry of Education and Culture, and the Cyprus National Bioethics Committee, 47 private and public nursery schools, and 69 primary schools in three provinces (Nicosia, Larnaca, and Limassol) were randomly selected for participation. Special Units operating within the schools did not participate in the study. All schools were informed about the aims of the study. School boards interested in participating in the study received details about the purpose and procedures via email or fax. Parents or guardians were informed about the nature of the study, and 81% consented to participate. Then, both fathers and mothers completed a package of questionnaires, which took approximately half an hour to be completed. No clinical samples or individuals with an official diagnosis of Autism Spectrum Disorder were included in the study.

### Measures

#### CU Traits

CU traits were measured using the 28-item parent report Child Problematic Traits Inventory (CPTI; Colins et al., 2014a, b). The CPTI captures three dimensions that mirror the adult three-factor model of psychopathy: grandiose-deceitful factor with eight items (Grandiose- Deceitful; e.g., “*Thinks that he or she is better than everyone on almost everything*”), callous-unemotional factor with ten items (Callous-Unemotional; e.g., “*Never seems to have a bad conscience for things that he or she has done*”), and impulsive-need for stimulation factor with ten items (Impulsivity – Need for Stimulation; e.g., “*Provides himself or herself with different things very fast and eagerly*”). For the current study, only the CU traits factor was used. Parents rated their children on a four-point Likert scale (from 1, “*Does not apply at all,*” to 4, “*Applies very well”*). Previous studies have verified that the CPTI total and factor scores demonstrate excellent reliability in terms of internal consistency, with αs raging between 0.89 and 0.96 in different samples (Colins et al., 2017; Wang et al., 2018). In the current study, the total CPTI factor (α = 0.92) and CU traits subscale demonstrated very good internal consistency (α = 0.85). Mothers’ and fathers’ reports were highly correlated (r = 0.60 for CU).

#### Autistic Traits

Autistic traits were measured using the school-age form of the Social Responsiveness Scale (SRS), a 65-item parent and/or teacher report (Constantino & Gruber, 2012). In the current study, SRS was used as a parent report. Parents rated their children on a 4-point Likert scale (0 = not true, 1 = sometimes true, 2 = often true, 3 = almost always true) with total scores ranging from 0 to 195, with higher scores indicating higher degrees of social impairment. SRS captures five domains/ treatment subscales of autistic traits: awareness, cognition, communication, motivation, and mannerisms. In the current study, only the total score of SRS was used. Previous studies have verified that SRS shows high internal consistency (α = 0.91–0.97) and acceptable inter-rater reliability (0.76 and 0.95) (Bölte et al., 2008; Constantino & Todd, 2003; Constantino et al., 2000). In the current study, the total SRS score demonstrated excellent internal consistency (α = 0.92). SRS was used for measuring autistic traits since it captures a continuous dimensional distribution of autistic symptoms and not a categorization distribution using a cut-off score.

#### Conduct Problems

The Eyberg Child Behavior Inventory (ECBI; (Eyberg et al., 1999) is a 36-item parent-rating scale of child conduct problems (e.g., lying, assaulting, engaging in fights). Parents indicate the intensity of the child’s behaviors on a seven-point Likert scale (from 1 ‘*never’* to 7 ‘*always’*). The Intensity score has a possible range between 36 and 252 and has demonstrated excellent internal consistency (α = 0.95; Eyberg et al., 1999), interrater (mother-father) reliability (α = 0.69; Eisenstadt et al., 1994), and test–retest reliability across 12 weeks (α = 0.80) and ten months (α = 0.75; (Funderburk et al., 2003). In the current study, total ECBI Intensity scores showed excellent internal consistency (α = 0.88). Mothers’ and fathers’ reports were highly correlated (r = 0.63).

#### Parental Distress

The Parenting Stress Index-Short Form (PSI-SF) is a 36-item parent-rating questionnaire that consists of three subscales: Parental Distress, Parent–Child Dysfunctional Interaction, and Difficult Child. In the current study, we were interested in measuring only parental distress. For this reason, only the 12-item parental distress subscale was used (e.g., “*Feel that I cannot handle things”; “Gave up my life for children’s needs”*). Parents rated each item on a five-point- Likert scale (from 1 *‘strongly disagree’* to 5 *‘strongly agree’*), with higher scores indicating greater stress levels. Previous studies have verified that the PSI-SF total (α = 0.90 and factor scores (Parental Distress; α = 0.81: Parent–Child Dysfunctional Interaction α = 0.89 Difficult Child; α = 0.88) demonstrate excellent reliability in terms of internal consistency (Aracena et al., 2016). In the current study, the Parental Distress factor showed excellent internal consistency (α = 0.87). Mothers’ and fathers’ reports were significantly correlated (r = 0.50).

#### Parental Care

The Parental Bonding Instrument (PBI: Parker et al., 1979) is a 25-item parenting-rating questionnaire consisting of two subscales: Parental Care with 12 items and Overprotection with 13 items. For the current study only, the items of parental care were included (e.g., *‘I speak to my child in a warm and friendly voice,’ ‘I appear to understand his/her problems and worries’*). Parents rated each item on a five-point Likert scale (from 1 *‘never’* to 5 *‘always’*), with higher scores indicating greater levels of parental care. Previous studies have verified that PBI demonstrates good reliability and validity, satisfactory construct, and convergent validity (Parker, 1989). In the current study, Parental Care subscale internal consistency was acceptable (α = 0.65). Mothers’ and fathers’ reports were significantly correlated (r = 0.45).

#### Parental Practices

Parental practices were assessed using the parent version of the Alabama Parenting Questionnaire (APQ; Frick, 1991). APQ is a 42-item questionnaire that captures five dimensions of parenting: parental involvement (e.g., *“You have a friendly talk with your mom/dad”*), positive parenting (e.g., *“Your parents tell you that you are doing a good job”*), poor monitoring (e.g., *“You stay out in the evening past the time you are supposed to be home”*), inconsistent discipline (e.g., *“ Your parents threaten to punish you and then do not do it”*), and corporal punishment (e.g., *“Your parents hit you with a belt”*). Ratings of the items are made on a 5-point Likert scale (1 = Never to 5 = Always), with a higher rating indicating greater levels of the specific parenting behavior. A previous study has verified the validity and reliability of APQ in community samples of Greek Cypriot children (Fanti & Centifanti, 2014). The current study used only four dimensions: parental involvement, positive parenting, inconsistent discipline (each variable with α = 0.70), and corporal punishment (α = 0.75).

### Plan of Analyses

First, correlation analyses were conducted in SPSS 28.0 to investigate the association between CU and autistic traits with parental dimensions (i.e., parental care, distress, and practices). We set the p-value at 0.01 to avoid Type I errors, corresponding to a 99% confidence interval. We performed a series of hierarchical multiple regression analyses with the predictors in each model being CU and autistic traits and the interaction of those two traits. Moreover, we controlled for age, sex and CPs in step 1 of the regression analysis. Dependent variables were parental care, distress and parental practices (positive parenting, involvement, inconsistent discipline and corporal punishment). In step 1, sex (0 = males; 1 = females), CPs and age were entered. In step 2, CU and autistic traits were entered. In step 3, the multiplicative two-way interactions were entered to test the interaction between the two traits. CU and autistic traits were centered by subtracting the sample mean from each participant's score. Next, the regression equation from the full sample was used to calculate the predicted values of the dependent variable at low (1 SD below the mean) and high levels (1 SD above the mean) of the predictors.

## Results

### Descriptive and Correlational Analyses

Descriptive statistics and zero-order correlations between CPs, CU and autistic traits, parental care, distress, and practices are reported in Table 1. Specifically, CPs, CU, and autistic traits were moderately positively correlated. Regarding CU traits, there was a moderate positive correlation with distress, inconsistent discipline, and corporal punishment, while there was a moderate negative correlation with parental care. Autistic traits were moderately positively correlated with parental distress and inconsistent discipline, while there was a moderate negative correlation with parental care.

**Table 1:** Correlations between conduct problems, callous-unemotional, parental care, distress, involvement, poor monitoring, inconsistent discipline, and corporal punishmen

	CU traits	Autistic Traits	CPs	Parental Care	Parental Distress	Parental Involvement	Positive Parenting	Inconsistent Discipline	Corporal Punishment		
	*r*	*r*	*r*	*r*	*r*	*r*	*r*	*r*		*Skewness*	*Kurtosis*
CU traits	-	.59^*^	.52^*^	-.47^*^	.35^*^	-.21	-.17	.37^*^	.33^**^	-.04	-.74
Autistic traits	-	-	.36^*^	-.27^*^	.32^*^	-.11	.01	.29^*^	.18	1.09	1.54
CPs	-	-	-	-.22	.13	-.03	.01	.30^*^	.38^**^	.11	-.36
Parental Care	-	-	-	-	-.37^**^	.45^*^	.42^**^	-.25^*^	-.24^*^	-.65	-.17
Parental Distress	-	-	-	-	-	-.27^*^	-.29^**^	.24^*^	.14	.04	-.10
Parental Involvement	-	-	-	-	-	-	.63^**^	-.04	-.02	-.65	.32
Positive Parenting	-	-	-	-	-	-	-	.14	.11	-.91	-.08
Inconsistent Discipline	-	-	-	-	-	-	-	-	.36^**^	.11	-.04
Corporal Punishment	-	-	-	-	-	-	-	-	-	.93	.30
*Descriptives*											
Mean	21.86	42.06	19.27	51.58	23.48	37.16	27.17	15.07	4.66		
SD	9.90	22.00	7.03	5.55	7.86	3.75	15.06	4.08	1.64		

Two-tailed significance: * entries are significant at *p* < .01

*CU* Callous-Unemotional, *CPs* Conduct Problems

### Hierarchical Multiple Regression Analysis

Hierarchical multiple regression analysis was performed to test the unique effects of CU and autistic traits on parental care, distress, and parental practices after controlling for the effects of CPs, sex and age. Tests to see if the data met the assumption of collinearity indicated that multicollinearity was not a concern (CPs, *Tolerance* = 0.66, *VIF* = 1.52; CU traits, *Tolerance* = 0.51, *VIF* = 1.98; autistic traits, *Tolerance* = 0.54, *VIF* = 1.85). Moreover, the scatterplot of standardized predicted values showed that the data met the assumptions of homogeneity of variance and linearity (see Appendix). Skewness and kurtosis values for autistic traits were moderately elevated, however, they fall within a moderate range that is considered acceptable (see Table 1).

#### CPs, Sex and Age

CPs, sex and age were entered at step 1 of the regression model to control the effects of both variables in parental care and distress (see Table 2) and parental practices (see Table 3). Entering CPs in step 1 revealed a significant negative association with parental care (*β* = *-0.24, p* < *0.01*) and significant positive associations with inconsistent discipline (*β* = *0.34, p* < *0.001*) and corporal punishment (*β* = *0.37, p* < *0.001*). In addition, analyses in Step 1 revealed that sex and age were not significant predictors for all cases. In step 2, after introducing the CU and autistic traits variables, relations between CPs and parental care (*β* = *0.02),* inconsistent discipline (*β* = *0.13*) and corporal punishment (*β* = *0.23)* did not remain significant (see Tables 2 and 3). A negative significant relation between age and parental distress was revealed in steps 2 and 3 (see Table 2).

**Table 2:** Relations between CU and autistic traits with parental care and distress

	Parental Care				Parental Distress			
	*B*	*SE*	*b*	*R*^*2*^	*B*	*SE*	*b*	*R*^*2*^
*Step 1*				.07				.03
Sex	-1.44	1.02	-.13		.72	1.47	.05	
CPs	-.19	.07	-.24^*^		.14	.10	.13	
Age	-.38	.35	-.10		-.60	.50	-.11	
*Step 2*				.22^**^				.20^**^
Sex	-.70	.96	-.06		-.08	1.37	-.01	
CPs	.01	.08	.02		-.16	.11	-.15	
Age	-.05	.34	-.01		-1.30	.48	-.24^*^	
Autistic traits	.01	.03	.02		.08	.04	.23	
CU traits	-.26	.06	-.47^**^		.26	.09	.34^**^	
*Step 3*				.23				.21
Sex	-.78	.95	-.06		-.06	1.36	-.01	
CPs	-.01	.08	-.01		-.14	.11	-.13	
Age	-.03	.34	-.01		-1.32	.48	-.24^*^	
Autistic traits	-.02	.03	-.06		.11	.04	.30^*^	
CU traits	-.24	.06	-.45^**^		.24	.09	.31^**^	
CU x Autistic traits	.01	.01	.15		-.01	.01	-.14	

Two-tailed significance: * *p* < .01; and ** *p* < .001 level

*CU* Callous-Unemotional,*CPs* Conduct Problems

**Table 3:** Relations between CU and autistic traits with parental involvement, positive parenting, inconsistent discipline and corporal punishment

	Parental Involvement				Positive Parenting				Inconsistent Discipline				Corporal Punishment			
	*B*	*SE*	*b*	*R*^*2*^	*B*	*SE*	*b*	*R*^*2*^	*B*	*SE*	*b*	*R*^*2*^	*B*	*SE*	*b*	*R*^*2*^
*Step 1*				.02				.03				.11^*^				.14^**^
Sex	.28	.71	.04		-.07	.52	-.01		.14	.71	.02		-.09	.28	-.03	
CPs	-.02	.05	-.04		.02	.04	.04		.19	.05	.34^**^		.08	.02	.37^**^	
Age	-.31	.24	-.12		-.31	.18	-.16		.03	.24	.01		-.04	.10	-.03	
*Step 2*				.05				.07				.19^*^				.18
Sex	.56	.72	.07		.16	.53	.03		-.25	.70	-.03		-.21	.28	-.07	
CPs	.05	.06	.09		.06	.04	.14		.07	.06	.13		.05	.02	.23	
Age	-.21	.25	-.08		-.29	.18	-.15		-.18	.24	-.07		-.08	.10	-.07	
Autistic traits	.01	.02	.03		.02	.01	.15		.01	.02	.08		-.01	.01	-.03	
CU traits	-.09	.05	-.24		-.08	.03	-.28		.12	.05	.31^*^		.04	.02	.25	
*Step 3*				.06				.07				.20				.18
Sex	.55	.72	.07		.16	.53	.03		-.26	.70	-.03		-.25	.28	-.07	
CPs	.04	.06	.08		.05	.04	.13		.06	.06	.12		.05	.02	.22	
Age	-.21	.25	-.08		-.29	.18	-.15		-.17	.24	-.06		-.08	.10	-.07	
Autistic traits	-.01	.02	-.02		.02	.02	.12		.01	.02	.02		-.01	.01	-.07	
CU traits	-.08	.05	-.22		-.07	.04	-.27		.13	.05	.33^*^		.04	.02	.27	
CU x Autistic traits	.01	.01	.09		.00	.00	.05		.01	.01	.10		.01	.01	.09	

Two-tailed significance: * *p* < .01; and ** *p* < .001 level

*CU* Callous-Unemotional, *CPs* Conduct Problems

#### CU and Autistic Traits with Parental Care and Distress

Regarding parental care, as shown in Table 2, entering CU traits in the model [*F* (4,117) = 8.58, *p* < 0.001, *R*^*2*^ = 0.22] showed a significant difference from step 1 to step 2 [∆*F* (2,117) = 11.90, *p* < 0.001, *∆R*^*2*^ = 0.15]. When CU traits were entered, the variables explained 15% of the variance, with the final model, including CPs, accounting for 22% of the variance. Concerning parental distress, entering CU traits in step 2 [*F* (4,119) = 5.38, *p* < 0.001, *R*^*2*^ = 0.20] showed a significant difference from step 1 [∆*F* (2,119) = 9.48, *p* < 0.001, *∆R2* = 0.17]. All in all, when CU traits were entered, the variables explained 17% of the variance, with the final model, including CPs, accounting for 20% of the variance. No significant direct association was revealed between autistic traits and parental care and distress. Moreover, entering interactions between CU and autistic traits in step 3 did not lead to any significant differences—thus, no significant two-way interaction was revealed (see Table 2).

#### CU and Autistic Traits with Parental Practices

Regarding inconsistent discipline, as shown in Table 3, entering CU traits in the model [*F* (4,117) = 6.77, *p* < 0.01, *R*^*2*^ = 0.19] showed a significant difference from step 1 [∆*F* (2,117) = 5.58, *p* < 0.01, *∆R*^*2*^ = 0.8]. When CU traits were entered, the variables explained 8% of the variance, with the final model, including CPs, accounting for 19% of the variance. No significant direct association was revealed between autistic traits and the four parental practices. Entering interactions between CU and autistic traits in step 3 did not lead to significant findings; thus, no significant two-way interaction was identified (see Table 3).

## Discussion

The purpose of this study was to explore whether there is a unique relation between CU and autistic traits with parental practices, care, and distress in a community sample. Additionally, the study aimed to examine the effects of the interaction of these two traits after controlling for confounding variables – CPs, age and sex. The research intended to address a gap in the literature, which has primarily focused only on examining the unique effects. One of the main findings of the study was that CU traits were significantly related to parental distress, practices and care, whereas autistic traits were not. Therefore, it appears that the behavioral and emotional characteristics associated with CU traits affect parents more strongly compared to autistic traits. Interestingly, the interaction between these two traits did not reveal any significant effect, pointing to the unique influence of CU traits on diverse parenting outcomes.

### CU Traits and Parental Practices, Care, and Distress

Our study partially supported our hypothesis that children with CU traits are more likely to experience negative and less likely to receive positive parental practices. These findings are consistent with prior research (e.g., Barker et al., 2011; Fanti et al., 2023), suggesting that revealed parents of children with high levels of CU traits tend to use inconsistent discipline, corporal punishment and express less care towards their children. This could be attributed to the behavioral and emotional characteristics of children with CU traits, such as callous use of others, lack of remorse, and empathic concern (Frick & White, 2008), which may result in frustration and harsh responses from parents (Trentacosta et al., 2019). Although this is not a longitudinal study, results may be partly related to the idea of a dynamic interaction between children and their environment, where temperamental factors associated with CU traits (i.e., fearlessness) may increase the likelihood of certain negative parental behaviors (Fanti et al., 2023). Moreover, our results indicated that this relation between CU and negative parental practices persists even after controlling for CP, proposing that the emotional affective component of CU traits plays a critical role in engaging in such parental behaviors above and beyond behavioral problems.

Focusing on positive parenting, results did not propose any association with CU traits. This contrasts with previous findings (Clark & Frick, 2018; Somech & Elizur, 2012), especially concerning the relationship with parental involvement. The absence of a negative association between positive parenting and CU traits in this study can be attributed to a few reasons. Firstly, positive parenting was considered a broad factor in this study. This approach is quite different from other studies that investigated specific components related to positive parenting, like warm affect, positive communication, and cooperation (Clark & Frick, 2018). Secondly, positive parenting appears to be strongly related to CPs rather than CU traits (Waller et al., 2013, 2014). Thus, positive parenting may be associated with CPs in children with heightened CU traits, rather than specifically CU traits. However, further research is needed to investigate this relationship. On the other hand, a negative relation between parental care and CU traits was revealed, indicating that parents of children with high levels of CU traits tend to exhibit behaviors that demonstrate lower levels of parental care. The unemotional profile of CU traits appears to be related to less expression of care from their parents. Again, these results can be linked to the idea that parenting is strongly affected by children’s characteristics (for a review, see Waller & Hyde, 2017).

The present study also demonstrated that parents of children with high levels of CU are related with heightened levels of parental distress, a finding that aligns with previous studies' results (e.g., Fanti & Centifanti, 2014; Fanti et al., 2017). Also, results showed that age became a significant factor only when CU traits were considered. This indicates that age affects the relationship between CU traits and parental distress, with younger ages being associated with higher levels of stress. One possible explanation is that parents tend to be more anxious and distressed about their children's behaviors during the preschool years, which tends to decrease as their children get older (Scheibe & Blanchard-Fields, 2009). Considering all findings, it is proposed that parents of children with high levels of CU traits might engage in more negative parental practices and express low levels of care as a consequence of their distress. Such practices may arise from a sense of dissatisfaction with the parental role or a feeling of hopelessness. Moreover, parents may perceive their CU children's affective characteristics as particularly distressing, leading to the adoption of more negative parenting practices as a coping mechanism. Parental distress can be both the cause and effect of negative parenting practices. As such, further research is required to clarify this potential bidirectional relationship. Lastly, no differences between boys and girls were found, indicating that sex does not impact the relations among the variables examined.

### Autistic Traits and Parental Practices, Care, and Distress

Findings did not support our hypothesis of a relation between negative parental practices and distress with autistic traits. Despite the general acceptance that social interaction and communication difficulties are major challenges for children with ASD or autistic traits in forming and maintaining positive relationships, including parents (Seltzer et al., 2000; Teague et al., 2017), our findings confirm that the relationship between parental dimensions and autistic traits is not yet clear. A possible explanation for the lack of association can be derived by the level of severity. Previous studies have highlighted that the level of severity of ASD and autistic traits is a crucial factor – since increased levels of severity lead to more severe communication and emotional expression deficits (Beurkens et al., 2013; Hoffman et al., 2009; Ku et al., 2019). In the current study, we used a community instead a clinical sample (i.e. children diagnosed with ASD) with probably less severe difficulties and deficits. Also, the age of participants was relatively young, an event which may lead parents to justify their children’s difficulties as a result of their young age or childhood immaturity rather than an indication of specific deficits.

Regarding parents’ distress, the explanation can be similar to the level of severity. In a recent review, Enea and Rusu (2020) propose that sensory and behavioral issues are the strongest predictors of parenting distress. Also, various studies have indicated that several factors influence parental distress. These factors include the child and parent characteristics, the knowledge the parents have regarding their child’s difficulties, and access to social and financial support and services (Enea & Rusu, 2020; Jacobs et al., 2020; Operto et al., 2021; Zablotsky et al., 2013). Therefore, moving to studies that focus on the dynamic association between individual and environmental factors may give us more concrete answers and help us fill the existing gaps in contradicting evidence. Another explanation can be derived from the young child’s age in the current study. Previous research proposes that parents experience greater challenges in family management as their children grow older, and also older age appears to be related to children's lower adaptive functioning (Operto et al., 2021; Tillmann et al., 2019). Therefore, the absence or relation between negative parental practices and distress can partly be attributed to low levels of severity, controlling the effects of CPs and children’s age. Again, no differences between boys and girls and age were found.

### Unique Associations and Interaction Effects

Contrary to our expectations, results did not reveal any interaction between autistic and CU traits with parental practices, care and distress. As far as we know, there are now previous studies investigating this concept, so findings should be interpreted with conscious. This lack of association can be further supported by the absence of any unique association between autistic traits and these variables after accounting for levels of CP and CU traits. Notably, at the zero-order level, autistic traits were significantly correlated with parental distress, inconsistent discipline, and parental care. However, when we added CU traits to the analysis, this relation disappeared, and no interaction was revealed, proposing the unique effects of CU traits on the outcome variables. As mentioned before, probably the level of severity (Enea & Rusu, 2020) is the factor that may be related to difficulties and may interact with CU traits in creating a “double-hit”. It has been observed that individuals with autistic traits can also display higher levels of CU traits (Chang et al., 2021; Leno et al., 2015; Rogers et al., 2006). However, this association is linked to less severe antisocial behavior. This may be due to the increased challenges faced by such individuals in socializing and interacting with others, which can lead to a lack of engagement with peers – thus, fewer changes for engaging in antisocial behaviours, as suggested by previous studies (Leno et al., 2015, 2021). Therefore, focusing on the study’s design, the absence of interactions may be due to the utilization of a non-clinical population. However, we decided to use a community sample since the clinical presentation of symptoms associated with ASD is characterized by a high degree of heterogeneity. Recent studies have provided evidence for a broad range of severity in autistic symptoms, which suggests a spectrum where some individuals may exhibit autistic traits without necessarily fulfilling the criteria for an ASD diagnosis (Constantino & Todd, 2003; Hill & Frith, 2003). Thus, by investigating the manifestation of these traits in the community, we might be able to understand the deficits that place children at risk for behavioral maladjustment and inform prevention programs.

### Strengths and Limitations

The current study has some strengths and limitations that should be noted. Among the strengths is the use of questionnaires appropriate for young children and the almost even representation of boys and girls. An additional strength is the investigation of interactions among CU and autistic traits in predicting parental practices, care and distress since, as far as we know, no prior studies have focused on such associations. Despite its strengths, our study has several limitations that must be considered when interpreting the findings. Our assessment of the constructs of interest was based on parent reports. Future research may benefit from using children's and teachers' or significant others' reports as well to enrich the understanding of parents' behaviors. However, since the current study focused also on preschool-aged children, it was deemed appropriate not to include children's reports. Secondly, our current analysis solely focuses on parental distress levels and does not consider additional factors that may impact stress, such as the parents' traits, awareness of their child's situation, and social and economic resources. We recommend that future investigations incorporate these variables for a more comprehensive understanding of parental distress. Finally, although several participants in our study showed elevated CU and autistic traits, we did not include individuals who met the clinical criteria for Autism Spectrum Disorder (ASD) or Limited Prosocial Emotion, as defined by the DSM-5. The use of a community sample may explain why no association between autistic traits and interactions with target variables was revealed. In light of this, it may be advantageous for forthcoming investigations to utilize a clinical sample.

## Conclusion

In conclusion, the current study aimed to investigate the relation between CU and autistic traits with parental distress, care and practices. Findings revealed that children with CU traits were more likely to experience negative parenting, while parents showed heightened levels of stress. Notably, the study did not find any association between CU traits and positive parental practices, which were mainly influenced by levels of CP. Further analysis indicated no significant relation between autistic traits and interactions with the target variables, signifying that these traits are not associated with difficulties in parenting and distress. However, it is imperative to note that the small community sample used in this study may reflect low levels of severity of autistic traits as opposed to the case where a clinical sample would be used, which may have influenced the outcomes. Overall, our study provides valuable insights into the impact of CU traits, suggesting that the unemotional profile may lead to increased parental distress and engagement in more negative parental practices. Thus, in the community setting, the presence of CU traits may signal a higher risk of parental distress and negative parenting practices. To address this issue, interventions are recommended to prioritize enhancing parental roles and developing more effective parenting practices as opposed to exclusively addressing the emotional and behavioral challenges of the child. It is proposed that a systemic approach that targets both parents and children should be implemented. Such an approach will serve to strengthen the sense of parental role and enhance positive parental practices, ultimately leading to better outcomes for parents and their children.

## Appendix

Hierarchical multiple regression analysis with Parental Care as the dependent variable.

Hierarchical multiple regression analysis with Parental Distress, as the dependent variable.
